# Comparison of Rapidly Proliferating, Multipotent Aortic Valve-Derived Stromal Cells and Valve Interstitial Cells in the Human Aortic Valve

**DOI:** 10.1155/2019/7671638

**Published:** 2019-09-10

**Authors:** Yuming Huang, Kang Xu, Tingwen Zhou, Peng Zhu, Nianguo Dong, Jiawei Shi

**Affiliations:** Department of Cardiovascular Surgery, Union Hospital, Tongji Medical College, Huazhong University of Science and Technology, Wuhan 430022, China

## Abstract

Aortic valve calcification is a common clinical disease, caused by valve interstitial cells (VICs), which initiate the thickening and then calcification of valve leaflets. Classical valve-derived cells can be seen in different cell populations according to their different morphologies, but it is not clear whether different types of mesenchymal cells exist. In this study, culture conditions for mesenchymal stromal cells were used to selectively isolate valve-derived stromal cells (VDSCs). After subculturing, the morphology, proliferation, multidifferentiation, immunophenotype, and gene expression profiling in isolated VDSCs were compared with those in conventional cultured VICs. VDSCs isolated from human aortic valves were uniform spindle-shaped fibroblasts, had mutilineage differentiation abilities, and proliferated faster than VICs. Classic mesenchymal markers including cluster of differentiation 90 (CD90), CD44, and CD29 were positively expressed. In addition, the stem cell markers CD163, CD133, and CD106 were all expressed in VDSCs. RNA-sequencing identified 1595 differentially expressed genes between VDSCs and VICs of which 301 were upregulated and 1294 were downregulated. Valvular extracellular matrix genes of VDSCs such as collagen type 1, alpha 1 (COL1A1), COL1A2, and fibronectin 1 were abundantly expressed. In addition, runt-related transcription factor 2 and Ki-67 proteins were also markedly upregulated in VDSCs, whereas there was less expression of the focal adhesion genes integrin alpha and laminin alpha in VDSCs compared to VICs. In conclusion, novel rapidly proliferating VDSCs with fibroblast morphology, which were found to express mesenchymal and osteogenic markers, may contribute to aortic valve calcification.

## 1. Introduction

Aortic valve stenosis is one of the most common cardiovascular diseases. Its prevalence is only about 0.2% in adults between the ages of 50 and 59 years but increases to 9.8% in octogenarians, with an overall prevalence of 2.8% in adults older than 75 years [1]. Many factors contribute to the pathogenesis of aortic stenosis such as congenital bicuspid valve and rheumatic heart disease, but the main cause is calcification [2]. Calcific aortic valve disease (CAVD) is an active pathobiological process at the cellular and molecular levels, which involves fibrosis and calcification of aortic valve leaflets causing hemodynamic changes in the heart and eventually contributes to heart failure [3]. CAVD is hypothesized to reach a “point of no return” beyond which pharmaceutical intervention is unlikely to stop or even slow its progression, and surgery may be the only option.

Mesenchymal stromal/stem cells (MSCs) were first identified by Friedenstein, who described an adherent fibroblast-like population from the bone marrow (BM), which could differentiate into the bone that he referred to as osteogenic precursor cells. Subsequent studies have demonstrated that these cells have multilineage differentiation capacity [4] and can migrate to various organs in the context of tissue remodeling, thereby representing a source of pluripotent cells for the repair of damaged tissue [5]. Although MSCs were originally isolated from BM, similar populations have been isolated from other tissues including adipose tissue, placenta, amniotic fluid, and fetal tissues such as fetal lung and the blood and even adult tissues such as the Achilles tendon, skin, and teeth [6, 7]. Recently studies have focused on the role of MSCs in disease and treatment, because of their differentiation potential and immunoregulatory capacity [8, 9].

The normal aortic valve is primarily populated by valvular interstitial cells (VICs), a heterogeneous, multipotent cell population responsible for maintaining valve homeostasis [10, 11]. Multiple cell types such as fibroblasts or smooth muscle cells and myofibroblasts contribute to this population. The aortic valve is rich in mesenchymal progenitor cells, which have a strong potential to contribute to valve calcification [12]. It has also been found that the recruitment of BM-derived VICs is a normal homeostatic process in mouse models of BM transplantation [13]. Moreover, circulating endothelial progenitor cells with an osteoblastic phenotype seem to contribute to aortic valve calcification [14].

The functions of various VIC subpopulations remain unclear. Thus, this study evaluated one subpopulation of VICs. For the first time, a similar culture protocol as that used for BM-MSCs was used to isolate valve-derived stromal cells (VDSCs) from human aortic valves. Then, these cells were compared to VICs with regard to proliferation, differentiation, immunophenotype, and differences in transcription.

## 2. Materials and Methods

### 2.1. VIC and VDSC Isolation and Culture

Valves were obtained from patients presenting with CAVD who gave written informed consent. The study was approved by the Ethics Committee of Tongji Medical College, Huazhong University of Science and Technology (Wuhan, China). Aortic valve leaflets were excised and rinsed according to our previous protocol [15]. Then, tissues were minced and placed in collagenase (150 units/mL) in Dulbecco's Modified Eagle's Medium (HyClone, Logan, UT, USA) for 6–8 h at 37°C. After collagenase digestion, the cell suspension was obtained by removing undigested tissue pieces with a 70 *μ*m cell strainer. Then, the cells were divided into two different media: (1) VICs were cultured in standard DMEM with 10% heat-inactivated FBS (Thermo Fisher Scientific, Waltham, MA, USA) and 150 U/mL penicillin/streptomycin (HyClone) and (2) VDSCs were cultured in human MSC complete medium (STEMCELL Technologies, Vancouver, British Columbia, Canada), with 2 mmol/L L-glutamine. VICs and VDSCs were seeded at 10,000 cells/cm^2^ in tissue culture flasks in complete medium, which was changed every 3 days, until VICs were about 90% confluent.

### 2.2. FCM

Different cell-surface markers were assessed via FCM. For this purpose, VICs (1 × 10^5^) were resuspended in 100 *μ*L phosphate-buffered saline and incubated for 30 min on ice with conjugated antibody against cluster of differentiation 29 (CD29), CD44, CD90, CD106, CD117, CD133, CD163, CD146, CD34, CD31, CD11b, and CD68 (all from BD Biosciences, Franklin Lakes, NJ, USA). Then, cells were fixed in 4% paraformaldehyde and washed twice.

### 2.3. Immunofluorescence

Cells were stained via immunofluorescence (IF) for the following markers: alpha smooth muscle actin (*α*-SMA; Boster, Wuhan, China), vimentin (Boster), Sry-related HMG box gene 10 (SOX-10; Abcam, Cambridge, MA, USA), rhodamine phalloidin (Cytoskeleton Inc., Denver, CO, USA), and Ki-67 (Cell Signaling Technology (CST), Danvers, MA, USA). VICs seeded on 48-well plates at a density of 5000 cells/well were washed twice with PBS and fixed in 4% paraformaldehyde for 10 min. The fixative solution was removed by rinsing three times with PBS. Cells were permeabilized with 0.2% Triton X-100 for 5 min, washed three times with PBS, and blocked for 30 min with goat serum albumin (Boster). Immediately after blocking, cells were incubated with primary antibodies at 4°C overnight. After washing three times with PBS, samples were incubated with secondary antibodies (CST) in PBS for 60 min at room temperature. Then, samples were washed twice with PBS and incubated with DAPI (BioFroxx GmbH, Einhausen, Germany) for 4 min to stain the nuclei. Samples were washed twice with PBS and then imaged on the Axio Observer Z1 microscope (Zeiss, Oberkochen, Germany).

### 2.4. *In Vitro* Multipotent Differentiation

To evaluate the trilineage differentiation of VDSCs and VICs, cells were harvested and plated in 6-well plates at a density of 2 × 10^4^ cells/cm^2^. For osteogenic and adipogenic differentiation, cells were cultured in the medium until they were 80–90% confluent. Then, the medium was replaced with osteogenic induction medium (ScienCell Research Laboratories Inc., Carlsbad, CA, USA) or adipogenic induction media (STEMCELL Technologies). To differentiate VICs and VDSCs into chondrocytes, the human MSC chondrogenic differentiation medium (STEMCELL Technologies) was used according to the manufacturer's instructions. Cells were cultured for 21 days with media changes every 3 days. VICs and VDSCs were cultured in DMEM with 2% FBS or human MSC base medium (STEMCELL Technologies) during the trilineage protocol as a negative control. Successful differentiation was evaluated by staining the differentiated cells with Oil Red O and Alizarin Red in cases of differentiated adipocytes and osteocytes, respectively. The pellets were paraffin-embedded using standard methods, and 6 *μ*m sections were stained with Alcian blue and Nuclear Fast Red or hematoxylin and eosin (H&E).

### 2.5. FCM Analysis of the Cell Cycle

VICs and VDSCs (passage 2) were cultured in 60 mm dishes until 80% confluency, after which, the medium was changed to DMEM with 2% FBS or MSC base medium for 8 h. Both cell lines were trypsinized and then resuspended in PBS at 5 × 10^5^/mL, followed by fixation in 70% precooled ethanol overnight at 4°C, centrifugation, washing, and staining with PI/RNase staining buffer (BD Biosciences) for 30 min at 4°C. Cell counts at different phases of the cell cycle were analyzed by FCM as previously described [16].

### 2.6. Cell Viability Assay

Cell viability was assessed with the Cell Counting Kit-8 (CCK-8) assay (http://Bimake.com, Houston, TX, USA) according to the manufacturer's instructions. The cells were seeded at a density of 5000 cells/well in 24-well plates and cultured for 1–6 days. At the end of each time interval, cell samples were washed with PBS and incubated with serum-free medium containing 10% CCK-8 reagent. After 4 h of incubation at 37°C in an atmosphere of 5% CO_2_, aliquots were pipetted into a 96-well plate and measured at 450 nm using an enzyme-labeling instrument (Thermo Fisher Scientific).

### 2.7. RNA-Sequencing of VICs and VDSCs

RNA-sequencing (RNA-seq) was utilized to compare the mRNA profiles between VICs and VDSCs. Isolated RNA was sent to BGI Tech Solutions Co. Ltd. (Shenzhen, China) for RNA-seq, which was performed on the BGISEQ-500 sequencer; all samples were sequenced in triplicate for confirmation purposes. Sequencing results were analyzed using the “R Project (version 3.5.1)” to identify differentially expressed genes (DEGs). Gene Ontology (GO) and Kyoto Encyclopedia of Genes and Genomes (KEGG) pathway enrichment analyses were also performed.

### 2.8. Statistical Analysis

RNA-seq results were analyzed using the R (version 3.5.1) according to a previous study [15], and all other data were analyzed and expressed as the mean ± standard deviation (SD). Statistical comparisons were made by analysis of variance to evaluate differences among groups. A *p* value less than 0.05 was considered statistically significant.

## 3. Results

### 3.1. Cell Morphology of VDSCs Evidently Differ from VICs

**Figure 1:**
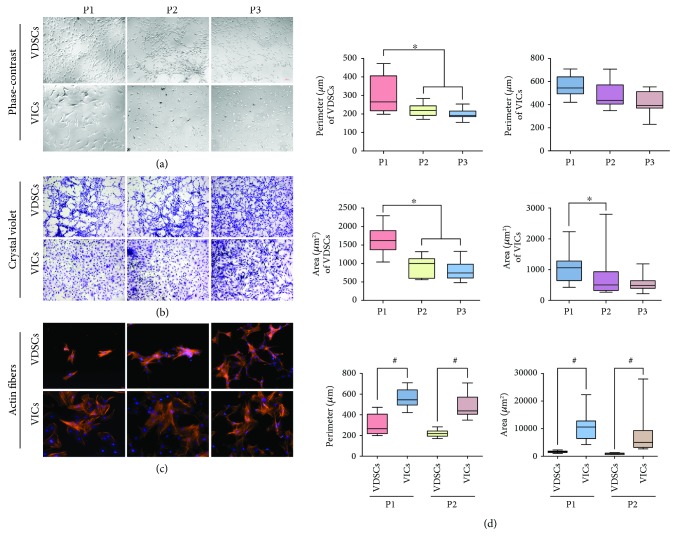
Cell morphology comparison between valve-derived stromal cells (VDSCs) and valve interstitial cells (VICs). (a) Phase-contrast images of VDSCs and VICs (passages 1 to 3: P1 to P3), (b) VDSCs and VICs with crystal violet staining, and (c) immunofluorescence staining of cellular actin stress fibers with rhodamine phalloidin and nuclei were stained with DAPI. (d) Quantitation of cell area (*μ*m^2^) and cell perimeter (*μ*m) comparison between VDSCs and VICs, ^∗^^,^^#^*p* < 0.05 are accepted as have significant difference, *n* = 12.

With culturing, the morphology of the VDSC was quite different from that of the classic VICs, which were like fibroblasts. The VICs had various morphologies including large and flat or small and flat and large spindle or small spindle, which means they belong to multiple cell populations. We compared passages 1–3 of VDSCs and VICs using phase-contrast or crystal violet-stained images (Figures 1(a) and 1(b)). Cell perimeters and areas were calculated (Figure 1(d)). With culturing from passages 1 to 3, the perimeter and area of VDSCs in passages 2 and 3 (P2 and P3) significantly decreased compared to P1 (^∗^*p* < 0.05). Compared to VICs, the VDSCs were much smaller regardless of the perimeter and area (^#^*p* < 0.05). Actin stress fibers stained with rhodamine phalloidin also showed a difference in the spreading areas of the cells (Figure 1(c)).

### 3.2. Comparison of Cell Proliferation between VDSCs and VICs

**Figure 2:**
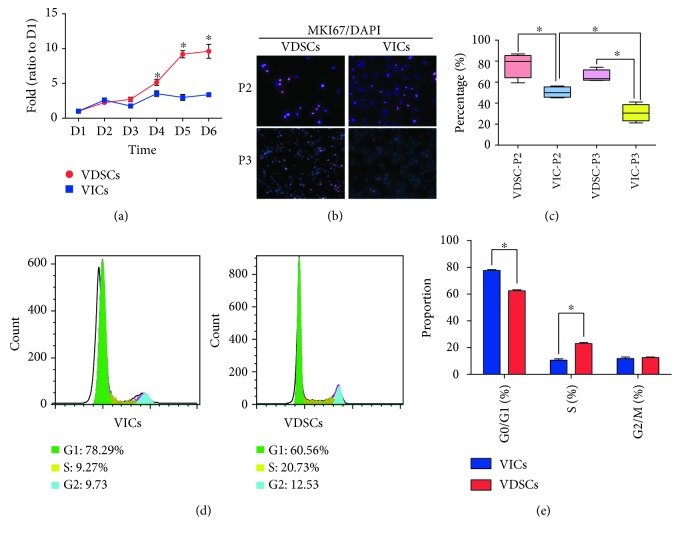
Cell proliferation ability comparison between VICs and VDSCs. (a) Cell proliferation curves show the difference between VDSCs and VICs; ^∗^*p* < 0.05 (vs. VICs) are accepted as having significant difference, *n* = 3. (b) MKI67 immunofluorescent staining, and nuclei are stained with DAPI. (c) The cartogram (based on (b)) shows the differences between passage 2 and passage 3 of VDSCs and VICs; ^∗^*p* < 0.05 are accepted as having significant difference, *n* = 4. (d) FACS analysis for the cell cycle of VDSCs and VICs; (e) S, G0/G1, and G2/M phases were counted and statistically compared. ^∗^*p* < 0.05 (vs. VICs) are accepted as having significant difference, *n* = 4.

Cell viability analysis of VDSCs and VICs up to 6 days showed that VDSCs proliferated faster than VICs during that time (Figure 2(a)). Compared with VICs, the proliferation rate of VDSCs exhibited significant differences on days 4, 5, and 6. MKI67 IF staining of both types of cells showed that about 80% of VDSCs were MKI67-positive at passage 2, which decreased to about 60% at passage 3, whereas about 50% of VICs were MKI67-positive at passage 2, and only about 30% were in passage 3 (Figures 2(b) and 2(c)). When VICs were compared to VDSCs at both passages 2 and 3, there was a significantly higher percentage of MKI67-positive VDSCs than VICs (^∗^*p* < 0.05). Cell cycle analysis by FCM revealed that the percentage of VDSCs in the S phase was about 20%, which was double the percentage of VICs (10%) (^∗^*p* < 0.05), whereas there were significantly fewer VDSCs in the G1 phase (60%) compared to VICs (80%) (^∗^*p* < 0.05; Figures 2(d) and 2(e)).

### 3.3. Different Immunophenotypes and Mutilineage Differentiation Ability of VDSCs and VICs

**Figure 3:**
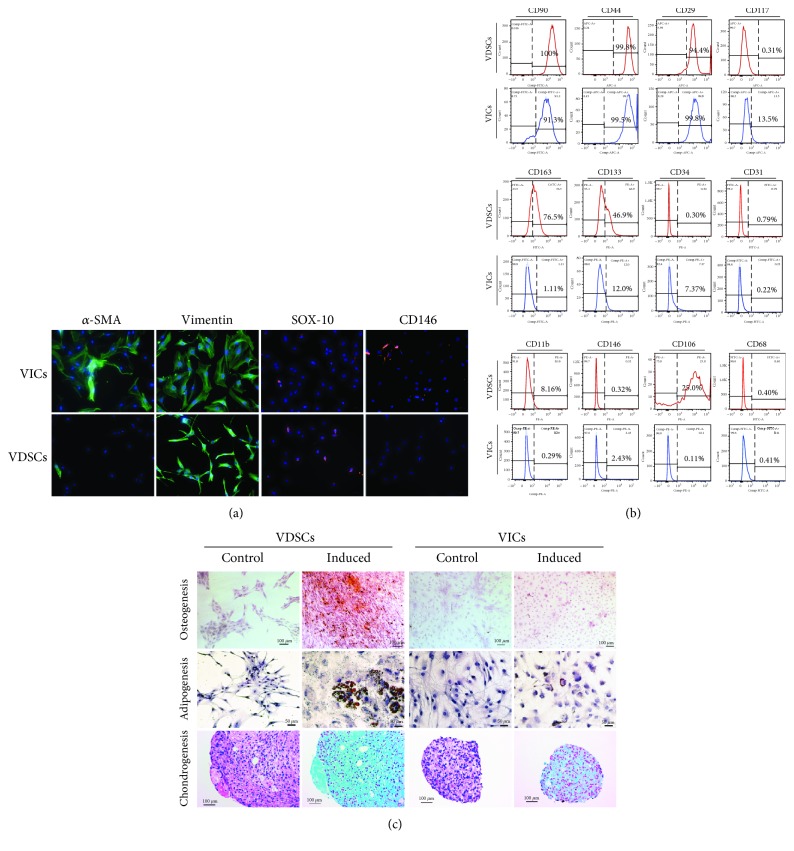
Comparative analysis of immunophenotype and differentiation abilities on VDSCs and VICs. (a) Immunofluorescent staining for *α*-SMA, vimentin, SOX-10, and CD146 of VDSCs and VICs. Nuclei are stained with DAPI; scale bars: 50 *μ*m. (b) Flow cytometric analysis of mesenchymal markers of CD90, CD44, CD29, CD117, CD163, CD133, and CD146 and endothelial markers of CD34, CD31, and CD106 and also hematologic markers of CD11b and CD68 in VDSCs and VICs. (c) Osteogenic, adipogenic, and chondrogenic comparison of the VDSCs and VICs (Alizarin Red S staining for osteogenesis, Oil Red O staining for adipogenesis, hematoxylin staining for the nuclei, and Alcian blue for chondrogenesis); scale bars: 100 *μ*m.

Due to the different morphologies and viabilities of VDSCs and VICs, we further evaluated some standard VIC markers via IF and FCM in cells at passage 3. The IF results (Figure 3(a)) showed that VDSCs were mostly negative for *α*-SMA, whereas VICs were mostly positive for *α*-SMA. VDSCs and VICs were all positive for vimentin but had different cytoskeleton morphologies, which were consistent with the results of rhodamine phalloidin staining. VDSCs and VICs were both partially positive for SOX-10. VDSCs were mostly negative for CD146. FCM surface markers revealed that VDSCs and VICs are all mostly positive for CD90, CD44, and CD29 (mesenchymal markers) and were mostly negative for CD34 and CD31 (endothelial markers), CD11b (hematologic marker), CD68 (macrophage), CD146, and CD117 (stem cell markers). VDSCs were relatively positive for CD163, CD133, and CD106 (surface markers) (Figure 3(b)). According to the differentiation-inducing experiments conducted in VICs and VDSCs, the multilineage differentiation potential of VDSCs was stronger than that of VICs (Figure 3(c)). After culturing with the same differentiation-inducing medium for 21 days, VDSCs had more calcium nodules and larger lipid droplets, as detected by Alizarin Red S and Oil Red O staining. VDSCs and VICs were pelleted and grown in chondrogenic media for 28 days. The pellets of VDSCs stained positive for Alcian blue, indicating the presence of proteoglycans for chondrogenesis.

### 3.4. Gene Expression Profiles Reveal Global Differences between VDSCs and VICs

**Figure 4:**
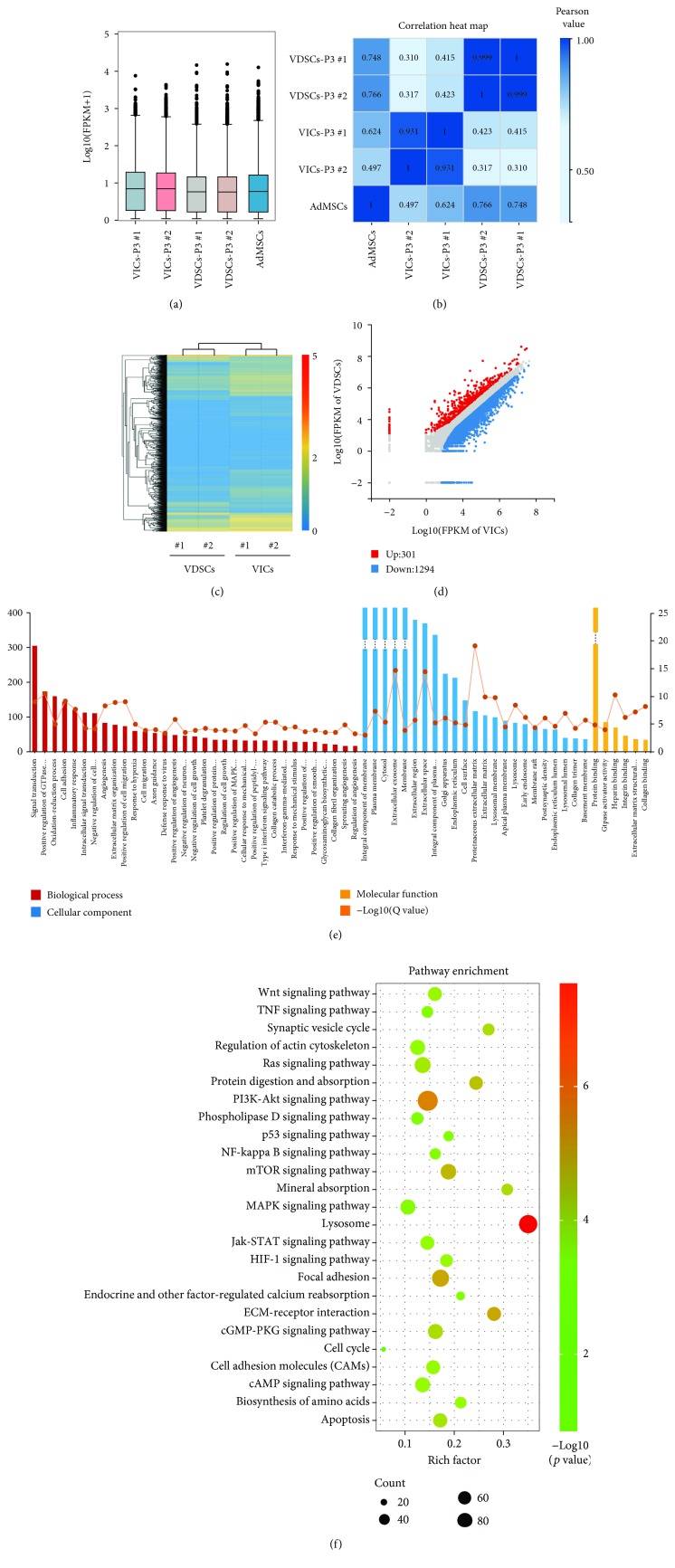
Comparison of global gene expression profiles between VICs and VDSCs. (a) Box plot shows the distribution of gene expression levels in each sample, and the dispersion of the data distribution can be observed; adipose mesenchymal stem cells (AdMSCs) were used for the reference. (b) Heat map for the Pearson correlation coefficient of all gene expression levels between samples; the higher the correlation coefficient indicates, the more similar the gene expression level. (c) Heat map for the global gene expression with group clusters (*n* = 2). (d) Scatter plot of differentially expressed genes (DEGs) in VDSCs versus VICs (upregulation: 301 and downregulation: 1294); FC (fold change) > 1 was accepted as positive DEGs. (e) GO enrichment of those selected DEGs including the biological process (red), cellular component (blue), and molecular function (yellow); broken line indicates *p* value (-log10). (f) KEGG pathways enrichment bubble map; a larger *p* value (-log10) indicates a higher degree of enrichment.

The box plot shows that the distribution of gene expression levels between VICs and VDSCs was scattered differently; the dispersion of the distribution of VDSCs was closer to that of adipose-derived MSCs (AdMSCs) (Figure 4(a)). The coefficient of gene expression levels revealed that VDSCs were highly different from VICs (#1: 0.310/0.415 and #2: 0.317/0.423) but were somewhat similar to AdMSCs (#1: 0.766 and #2: 0.748; Figure 4(b)). Heat map and global gene expression analysis showed two types of cluster for distinguishing between VDSCs and VICs (Figure 4(c)). A scatter plot of the DEGs (FC (fold change) > 1; *p* < 0.05) showed that 301 genes were upregulated and 1294 were downregulated between VDSCs and VICs (Figure 4(d)). KEGG pathway analysis was performed on the identified DEGs described above. Our results showed that these DEGs were highly enriched in functions related to lysosomes, phosphoinositide 3 kinase-Akt, mechanistic target of rapamycin, focal adhesion, extracellular matrix- (ECM-) receptor signaling pathways, and others (Figure 4(e)). Furthermore, GO functional annotations were made on the above-identified DEGs (1595 genes), as shown in Figure 4(f). Molecular function analysis indicated that some of the above DEGs were highly involved in protein binding. GTPase activator activity, and etc. These DEGs were enriched in the cellular component: plasma membrane, cytosol, and extracellular exosome, most of which are involved in signal transduction, positive regulation of GTPase activity, and oxidation-reduction processes (Figure 4(f)).

### 3.5. Analysis and Comparison of Selected Genes between VDSCs and VICs

**Figure 5:**
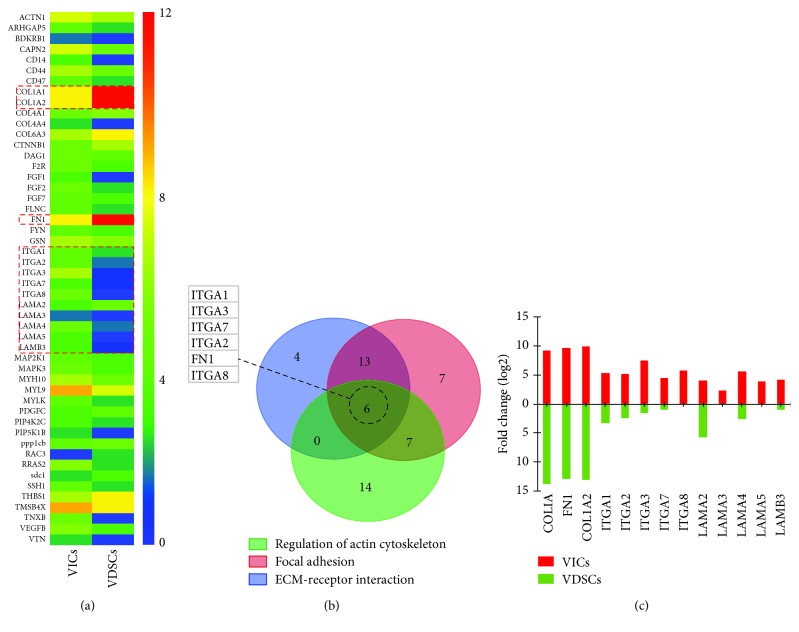
Significant pathways (ECM-receptor interaction, focal adhesion, regulation of actin cytoskeleton, and cell cycle) were selected for further significant DEG analysis. (a) Heat maps for typical selected functional DEGs based on previous selected pathways, (b) Venn interaction of DEGs, and (c) selected ECM-related DEG fold change in VICs and VDSCs.

To explore the significant differences in morphology between VDSCs and VICs, when combined with KEGG-enriched pathways, ECM-receptor interaction, focal adhesion, and regulation of actin cytoskeleton were selected for further DEG analysis to identify critical targets. Fifty-one common DEGs were selected based on the above-mentioned pathways concerning cell shape and spread (Figure 5(a)). After Venn interaction (Figure 5(b)), integrin alpha (ITGA)1, 2, 3, 7, and 8 and fibronectin 1 (FN1) were identified as associated with cell adhesion and migration. In addition, COL1A1, COL1A2, and FN1 gene expression levels in VDSCs were significantly higher than those in VICs, whereas levels of ITGAs and LAMAs were all lower than those of VICs (Figure 5(c)). In addition, the gene expression of runt-related transcription factor 2 (RUNX2), a recognized osteogenic marker, and MKI67 (cell proliferative marker) in VDSCs were markedly upregulated compared to VICs (Supplementary Data ([Supplementary-material supplementary-material-1]): fragments per kilobase of transcript per million mapped reads of RNA-seq).

## 4. Discussion

VICs cultured *in vitro* in 10% FBS-DMEM had various cell morphologies of different shapes and different sizes; when the valve endothelial cells (VECs) that totally differed from the interstitial cells are wiped out, diverse VIC subpopulations should exist. To the best of our knowledge, this is the first time MSC culture conditions were used to separate morphologically homogeneous VDSCs. These cells were much smaller in size regardless of the areas and the perimeter. The nuclear-cytoplasmic ratio of VDSCs was similar to that of MSCs. From a morphological aspect, one VIC subpopulation had a uniform morphology similar to that of MSCs.

MSCs have extremely strong proliferative ability [4, 17, 18]; they can establish clonal growth in a density-independent fashion. Our results showed that VDSCs have stronger proliferative ability than VICs. MKI67 IF staining of both types of cells showed that about 80% of the VDSCs were MKI67-positive at passage 2, which decreased to about 60% at passage 3, whereas about 50% of the VICs were MKI67-positive at passage 2, which decreased to only 30% at passage 3. When compared with VICs at passages 2 and 3, the percentage of MKI67-positive VDSCs was significantly higher than that of VICs. MKI67 is a nuclear protein that may be necessary for cellular proliferation and is used as a cellular marker of proliferation. The cellular content of Ki-67 protein markedly increases during cell progression through the S phase of the cell cycle [19, 20], which is consistent with our results. Cell cycle analysis by FCM revealed that the percentage of VDSCs in the S phase was about 20% compared to 10% of VICs. One important feature of aortic valve calcification is the excess production and disorganization of collagen fibers and other ECM proteins [21], possibly due to rapid cell proliferation and strong secretory capacity. Thus, VDSCs may have different and important roles from VICs in aortic valve disease.

Differentiation is another capacity of MSCs. Although VICs have multilineage differentiation potential [12], our study showed that VDSCs appeared to have stronger potential. After culturing in the same differentiation-inducing medium for 21 days, VDSCs had more calcium nodules and larger lipid droplets, as detected by Alizarin Red S and Oil Red O staining. After being grown in chondrogenic media for 28 days, obvious pellets were seen and stained positive by Alcian blue. The later propagation phase of aortic valve stenosis is where procalcific and proosteogenic factors take over and ultimately drive disease progression [22], so VDSCs definitely contribute to this process. The detection of FCM surface markers revealed that VDSCs and VICs had different expressions of CD163, CD133, and CD106. CD106, also known as vascular cell adhesion molecule 1, is a cell-surface protein involved in the adhesion of leukocytes to the vascular endothelium, which is also expressed in a fraction of MSCs. Previous studies have suggested that CD106^+^ human BM-MSCs showed higher clonogenic capacity, exhibited a faster growth rate and robust multilineage differentiation, and had stronger immune regulatory activity [23]. Therefore, CD106^+^ VDSCs exhibit more reactions, as one of the important triggers of calcific aortic valve disease is sterile and nonsterile inflammation [11]. CD133, also known as prominin-1, is a five transmembrane domain cell-surface glycoprotein that localizes to membrane protrusions. It is expressed in many stem cells or progenitor cells, although its precise function is still unclear [24]. Therefore, it can be used as a specific marker for valve progenitor cells. In addition, CD163 is a member of the scavenger receptor cysteine-rich superfamily, and its expression is restricted to the monocytic-macrophage lineage with high expression in, for example, red pulp macrophages, BM macrophages, liver macrophages (Kupffer cells), lung macrophages, and macrophages of several other tissues [25]. Interestingly, VDSCs were partially positive for CD163. This is an important finding that needs further investigation. It is implied that CD163^+^ VDSCs were probably derived from resident valve macrophages [26].

According to the RNA-seq results, coefficient of gene expression levels revealed that VDSCs were highly different from VICs (#1: 0.310/0.415 and #2: 0.317/0.423) but had some similarity to AdMSCs (#1: 0.766 and #2: 0.748), indicating that VDSCs have basic MSC gene expression profiles. In addition, the gene expression levels of ITGA1, 2, 3, 7, and 8; COL1A1; COL1A2; and FN1 in VDSCs were significantly higher than those in VICs. The gene expression of RUNX2 and MKI67 in VDSCs was markedly upregulated compared to VICs. This result also confirms the above-mentioned cytological differences between VICs and VDSCs.

In conclusion, novel, rapid proliferative VDSCs with fibroblast morphology, which were found to express mesenchymal and osteogenic markers, may serve as a novel target that contributes to aortic valve calcification.

## Data Availability

All data included in this study are available upon request by contact with the authors.

## Supplementary Materials

Supplementary Materials The excel file is the RNA-seq data of all the samples. In order to let the reviewers better understand our global gene expression profile comparison between two types of cells, we provide the original data of the RNA-sequencing.
